# Cyclisation mechanisms in the biosynthesis of ribosomally synthesised and post-translationally modified peptides

**DOI:** 10.3762/bjoc.12.120

**Published:** 2016-06-20

**Authors:** Andrew W Truman

**Affiliations:** 1 Department of Molecular Microbiology, John Innes Centre, Colney Lane, Norwich, NR4 7UH, UK

**Keywords:** biosynthesis, cyclisation, enzymes, peptides, RiPPs

## Abstract

Ribosomally synthesised and post-translationally modified peptides (RiPPs) are a large class of natural products that are remarkably chemically diverse given an intrinsic requirement to be assembled from proteinogenic amino acids. The vast chemical space occupied by RiPPs means that they possess a wide variety of biological activities, and the class includes antibiotics, co-factors, signalling molecules, anticancer and anti-HIV compounds, and toxins. A considerable amount of RiPP chemical diversity is generated from cyclisation reactions, and the current mechanistic understanding of these reactions will be discussed here. These cyclisations involve a diverse array of chemical reactions, including 1,4-nucleophilic additions, [4 + 2] cycloadditions, ATP-dependent heterocyclisation to form thiazolines or oxazolines, and radical-mediated reactions between unactivated carbons. Future prospects for RiPP pathway discovery and characterisation will also be highlighted.

## Introduction

Nature employs a number of routes to produce peptidic secondary metabolites, including non-ribosomal peptide synthetases [1–2] (NRPSs) and diketopiperazine-forming cyclases [3–4]. Alternatively, peptides synthesised by the ribosome can be post-translationally modified into secondary metabolites [5]. These are termed ribosomally synthesised and post-translationally modified peptides (RiPPs), and they are prevalent throughout nature. Massive advances in genome sequencing has revolutionised the discovery of new natural products from all biosynthetic classes [6–8], and it has been particularly beneficial for the discovery of new RiPP pathways, which are often small and lacking in homology to one another [9]. There has therefore been a massive increase in the study of their biosynthesis in recent years.

**Figure 1:**
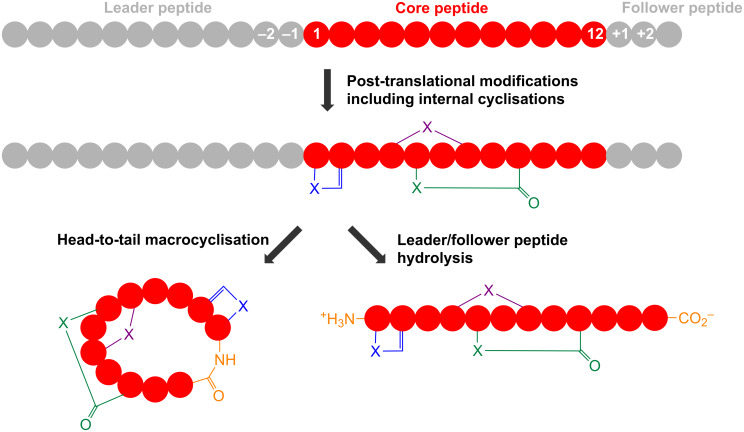
Schematic of RiPP biosynthesis. Thiazole/oxazole formation is represented by the blue heterocycle (X = S, O), lanthionine formation is represented by the purple cross-link (X = S) and macrolactam (X = N) or macrolactone (X = O) formation is represented by the green cyclisation.

**Figure 2:**
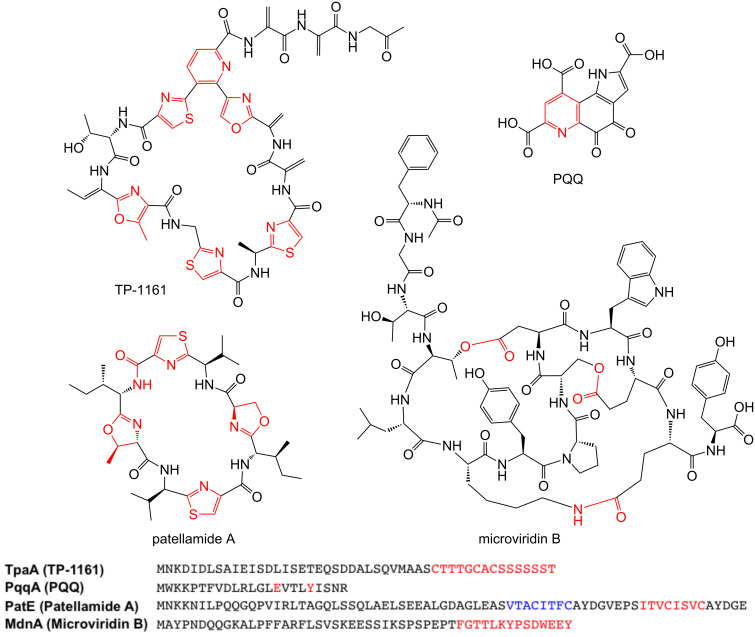
Examples of heterocycles in RiPPs alongside the precursor peptides that these molecules derive from. The red features on the molecules indicate where cyclisation has taken place, while the sections of the sequences highlighted in red correspond to the core peptides for each of these molecules. The sequence highlighted in blue in PatE corresponds to the core peptide for patellamide C, another macrocyclic RiPP that contains thiazoles and oxazolines.

RiPPs usually originate from a larger precursor peptide that consists of an N-terminal leader sequence and a core peptide that contains the natural product precursor (Figure 1). The bottromycin precursor peptide represents a notable exception as it features an N-terminal core peptide and a C-terminal follower peptide [10–13]. The core peptide is post-translationally modified and cleaved from the leader peptide to yield a biologically active peptide natural product (Figure 1 and Figure 2). A huge variety of RiPP post-translational modifications have been identified [5,14]; some are specific to certain classes of RiPP while others occur across the entire RiPP spectrum. These modifications can range from leader peptide hydrolysis and disulphide bond formation through to the complex remodelling of almost every amino acid in a molecule. For example, thiopeptide antibiotics [15] and the marine toxin polytheonamide [16] were both believed to be non-ribosomal peptides for a number of years, while the bacterial cofactor pyrroloquinoline quinone (PQQ, Figure 2) has a ribosomal origin [17] but has been modified so that no peptide bonds remain. This demonstrates that a huge amount of structural diversity can be introduced into RiPPs, despite an intrinsic requirement to be assembled from the 20 regular proteinogenic amino acids (possibly 21, as RiPPs containing selenocysteine were proposed in a recent bioinformatic study [18]). Excitingly, the ribosomal origin of RiPPs means that significant chemical changes to complex natural products can be achieved by simple site-directed mutagenesis. This requires the associated tailoring enzymes to tolerate a modified substrate, and there are many examples of pathways whose precursor peptides can be extensively mutagenised [19–23]. This is a powerful tool for the generation of natural product analogues and means that RiPP libraries can be generated much more rapidly and predictably than molecules made from multi-domain megasynthases such as polyketides and non-ribosomal peptides.

Cyclisation is a common post-translational modification in RiPP pathways and includes a multitude of transformations. These modifications are usually essential for the proper biological activity of the RiPP, as they fundamentally change the shape of a molecule, which can be critical for receptor binding or for protection from proteolysis. Examples include amide bonds, heterocyclisation to form thiazolines or oxazolines [24] (Figure 2), oxidative carbon–carbon bond formation [25] and thioether cross-links [26]. Fascinatingly, a significant number of these modifications are unique to RiPPs [27]. This review will focus on cyclisations that have been mechanistically characterised, as well as reactions where a mechanism can be confidently postulated. Disulphide bond formation is common in RiPP pathways but is found across proteins of all sizes so will not be discussed here.

## Review

### Thiazole and oxazoles

**Figure 3:**
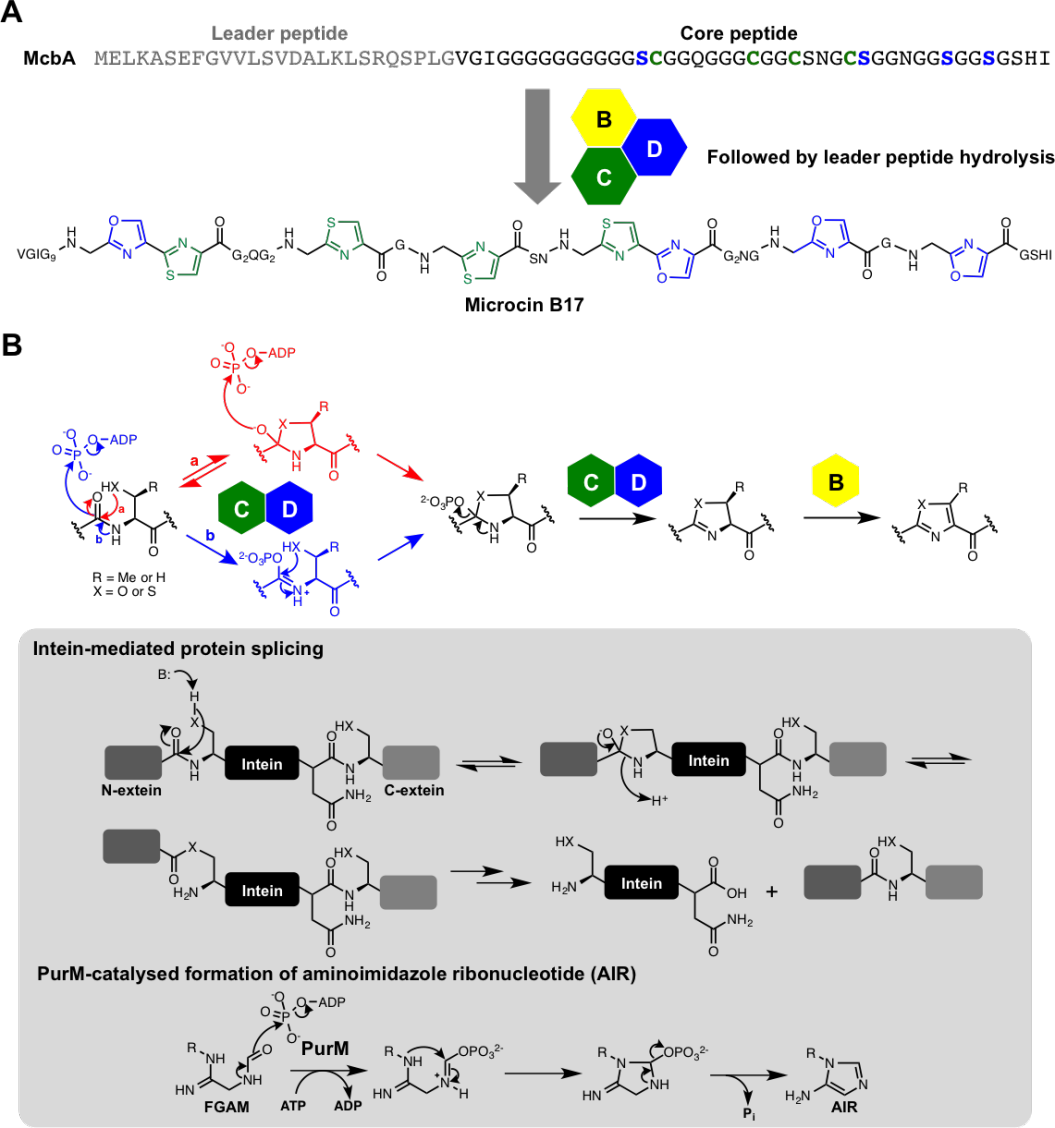
Formation of thiazoles and oxazoles in RiPPs. A) Biosynthesis of microcin B17. B) Mechanistic models for the introduction of azol(in)es into microcin, where pathway a was reported by the authors as the likely order of steps. An analogous mechanism was proposed in the biosynthesis of trunkamide, but with the transfer of AMP instead of phosphate. Inset: partial mechanism of intein-mediated protein splicing, which proceeds via a reversible hemiorthoamide, and the proposed mechanism of PurM-catalysed conversion of formylglycinamide ribonucleotide (FGAM) into aminoimidazole ribonucleotide (AIR), which involves activation of an amide by ATP and a 5-*endo*-trig cyclisation.

Thiazoles and oxazoles are found in a huge number of bacterial RiPPs, which are often loosely defined as thiazole/oxazole-modified microcins [24] (TOMMs), although these can be subdivided more accurately into a variety of structural classes, including linear azol(in)e-containing peptides (LAPs, e.g., microcin B17 [28], Figure 3A), thiopeptides (e.g., TP1161 [29], Figure 2) and cyanobactins [30] (e.g., patellamide A [31], Figure 2). In each class, the biosynthetic route to generate azol(in)es is highly similar, and is distinct from their generation in non-ribosomal peptides. The first in vitro reconstitution of a TOMM was carried out with microcin B17 [28,32–33], which showed that there are four essential proteins for its biosynthesis: the precursor peptide (the “A” protein McbA) that is post-translationally modified into the final product, and a heterotrimeric complex that is responsible for both heterocyclisation of serine and cysteine residues, and subsequent oxidation of (ox/thi)azolines into (ox/thi)azoles (Figure 3A). This catalytic complex consists of “C” and “D” proteins (annotated as McbB and McbD, respectively, for microcin B17) that cooperate to catalyse heterocyclisation of specific serine and cysteine residues in McbA, and a flavin-dependent dehydrogenase (the “B-protein”, McbC for microcin B17) that oxidises these heterocycles. These early in vitro studies indicated that the “C-protein” was a zinc-containing cyclase, and the “D-protein” possesses ATPase activity. The requirement for ATP turnover during cyclisation led to the hypothesis that the D-protein was a docking protein that regulates heterocyclase activity [33], while the presence of zinc in the C-protein pointed towards a catalytic role for this metal [33]. However, this role was later demonstrated to be structural rather than catalytic [34].

The Mitchell group showed [35] that the D-protein is actually directly involved in catalysis and uses ATP to activate the backbone amide bond for cyclodehydration, thus explaining the hydrolysis of ATP. A stoichiometric ratio of 1:1 between azole formation and ATP hydrolysis was demonstrated, and [^18^O]H_2_O was used in the reaction to show that oxygen incorporated into phosphate following ATP turnover was not derived from bulk water. This led to a mechanistic model where a reversible hemiorthoamide is first formed by side-chain *S*- or *O*-attack onto the amide carbonyl [35] (Figure 3B, pathway a), which is analogous to a step proposed for protein autoproteolysis [36] (Figure 3, inset). The exocyclic oxygen in this intermediate then attacks the α-phosphate of ATP to displace ADP and generate a phosphorylated hemiorthoamide. This highly reactive intermediate ensures that the rapid elimination of phosphate to generate (ox/thi)azolines is thermodynamically favourable. [^18^O]-labeled precursor peptide was subsequently used to further substantiate this proposal [37].

A similar heterocyclisation mechanism was proposed by the Naismith group for the cyanobactin heterocyclase TruD, which contains fused C- and D-proteins [38]. Interestingly, this revealed a notable difference with the microcin pathway, as cyclisation was accompanied by the generation of AMP and pyrophosphate (PP_i_), instead of ADP and phosphate. This points to an adenylation-type mechanism, and the authors also proposed a hemiorthoamide mechanism to account for the absence of wasted ATP hydrolysis (Figure 3B, pathway a). An alternative mechanism that would also account for the [^18^O]-labelling results involves direct activation of the amide carbonyl by ATP (Figure 3B, pathway b), which is analogous to a reaction catalysed by PurM family enzymes (aminoimidazole ribonucleotide synthetases) in the biosynthesis of aminoimidazole ribonucleotide as part of the purine biosynthetic pathway [39] (Figure 3, inset). This activated amide would then be attacked by an adjacent serine or cysteine side chain, thus releasing phosphate/AMP and generating the heterocycle. This order of steps was not advocated by either the Naismith or Mitchell groups as it requires a disfavoured 5-*endo*-trig cyclisation, although this mode of cyclisation is postulated to be catalysed by PurM, and Baldwin disfavoured cyclisations do occur in other biosynthetic pathways [40–41].

Curiously, members of the D-protein family are commonly annotated as YcaO domain proteins [35], where YcaO is an *E. coli* protein (Ec-YcaO) of unknown function that has been implicated in the β-methylthiolation of ribosomal protein S12 [42]. Crystallographic analysis has demonstrated that Ec-YcaO is structurally homologous to RiPP D-proteins and that ATP-binding residues are conserved across the superfamily [38,43]. Furthermore, biochemical studies showed that Ec-YcaO hydrolyses ATP to AMP and pyrophosphate [43]. The function of this highly conserved “non-TOMM” protein has yet to be identified, but it indicates that amide activation by ATP may not be confined to the biosynthesis of secondary metabolites or purines. Ec-YcaO also lacks a partner C-protein, which is also the case for a number of characterised secondary metabolite pathways. For example, the bottromycin gene cluster encodes two stand-alone YcaO domain proteins that have been postulated to participate in heterocyclisation reactions [10–13].

### Lanthionine bond formation in lanthipeptides

**Figure 4:**
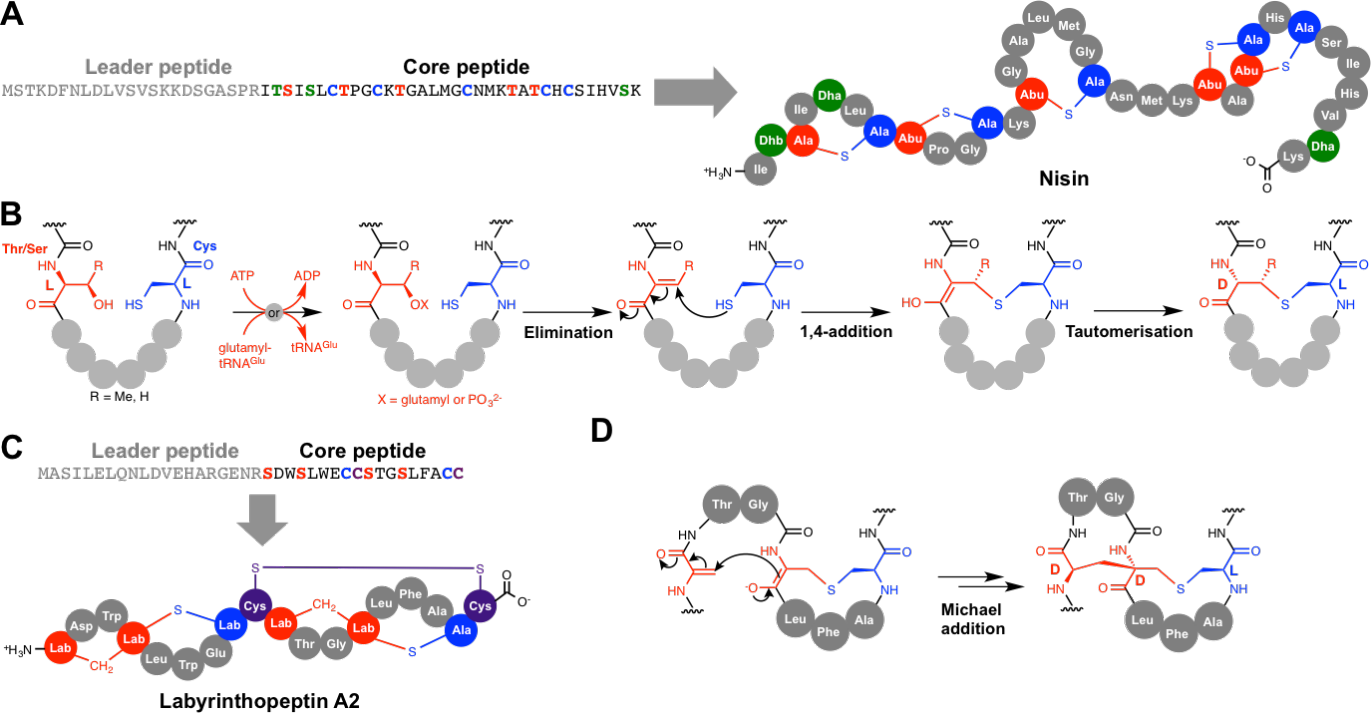
Lanthionine bond formation. A) Nisin and its precursor peptide. B) Mechanism of lanthionine bond formation for class I–IV lanthionine synthetases. GTP is used in an analogous way to ATP by some enzymes, for example in the biosynthesis of labyrinthopeptin A2. C) Labyrinthopeptin A2 and its precursor peptide. D) Mechanism for labionin formation in the biosynthesis of labyrinthopeptin A2.

Lanthipeptides (alternatively named lantipeptides [44]) are large bacterial RiPPs, and the first member to be reported was nisin (Figure 4A) from *Lactococcus lactis* in 1928 [45]. Many members of this family have antibacterial activity and these are termed lantibiotics [46]; nisin itself is used as a food preservative as it suppresses bacterial spoilage. Lanthipeptides are characterised by *meso*-lanthionine (Lan) and (2*S*,3*S*,6*R*)-3-methyllanthionine (MeLan) residues. Lanthionine consist of two alanine residues linked via a thioether that connects their β-carbons, while MeLan contains an additional methyl group (Figure 4B). These crosslinks are formed via a two-stage process. Firstly, serine (for Lan) and threonine (for MeLan) residues are dehydrated to 2,3-didehydroalanine (Dha) and (*Z*)-2,3-didehydrobutyrine (Dhb), respectively (Figure 4B). This is followed by 1,4-nucleophilic additions onto these didehydro amino acids by cysteine residues [47–49]. Lanthipeptides are divided into four distinct classes (I–IV) based on the differences between the biosynthetic enzymes that carry out dehydration and cyclisation [44]. Dehydration in class I lanthipeptide pathways is catalysed by a LanB dehydratase (NisB for nisin) and cyclisation is catalysed by a zinc-dependent LanC cyclase (NisC). In nisin biosynthesis, the precursor peptide, NisA, is dehydrated 8 times by NisB [50], and this has been shown to occur with directionality from the N- to C-terminus of the core peptide [51].

In vitro reconstitution of NisB activity with the nisin precursor peptide NisA showed that dehydration involves the glutamylation of Ser and Thr side chains prior to elimination of glutamate [50]. This mechanistic proposal was established due to the observation that three NisB mutants (R786A, R826A and H961A) were able to transfer multiple glutamates to NisA without subsequent elimination. Wild-type NisB was then able to convert polyglutamylated NisA to dehydrated NisA without the need for any additives that are usually necessary for NisB in vitro activity, thus demonstrating that glutamylated NisA is an authentic activated intermediate. Subsequent biochemical and structural work identified that glutamate is supplied by glutamyl-tRNA, and that glutamylation and elimination steps are catalysed by distinct domains within NisB [52]. Protein homology analysis indicated that LanB-like proteins are widespread in bacteria [52], so this unusual use of an aminoacyl-tRNA may actually be common across nature. Interestingly, a subset of these proteins lack the elimination domain and are commonly associated with NRPSs rather than RiPPs, but the function of these small LanBs is not yet known [52–53].

In contrast to class I lanthipeptides, both dehydration and cyclisation reactions are catalysed by bifunctional lanthionine synthetases for classes II–IV [47,49,54]. Furthermore, dehydration in each of these classes has been shown to proceed via phosphorylation of the amino acid side chain rather than by glutamylation [54]. Class II synthetases (“LanM”) have an N-terminal dehydratase domain and a C-terminal LanC-like cyclase domain, and detailed mechanistic studies on LamM enzymes was enabled by the in vitro reconstitution of lacticin 481 synthetase, LctM [47,54–56]. Both class III (“LanKC” [57]) and IV (“LanL” [49]) synthetases feature three domains, where a central kinase domain catalyses phosphorylation and an N-terminal lyase domain catalyses elimination [58]. Both class III and IV synthetases have C-terminal LanC-like cyclase domains, but class III enzymes lack the three conserved residues that bind zinc in the other classes [57], which is surprising, given that the active site Zn^2+^ is proposed to activate the cysteine side chains for cyclisation. The identification of the labyrinthopeptins [59] (Figure 4C) led to the discovery of a subset of class III lanthipeptides that contain an additional carbocyclic ring, which features the labionin (Lab) amino acid (Figure 4C). This is formed by sequential Michael-type cyclisations [57,60], where a conventional lanthionine thioether is first formed by the attack of cysteine onto Dha. The resulting enolate then attacks another Dha residue to stereospecifically form the carbocycle (Figure 4D), and the stereochemical outcome of this cyclisation is equivalent to lanthionine formation [59]. Both S–C and C–C crosslinks are formed by the same enzyme, LabKC, which also catalyses the formation of the Dha residues. An elegant experiment using a series of peptides with α-deuterated serine residues demonstrated that LabKC dehydrates the precursor peptide with C- to N-terminal directionality [61], which is in contrast to NisB from the nisin pathway, which processes its peptide in the opposite direction [51].

### Aminovinylcysteine-containing peptides

**Figure 5:**
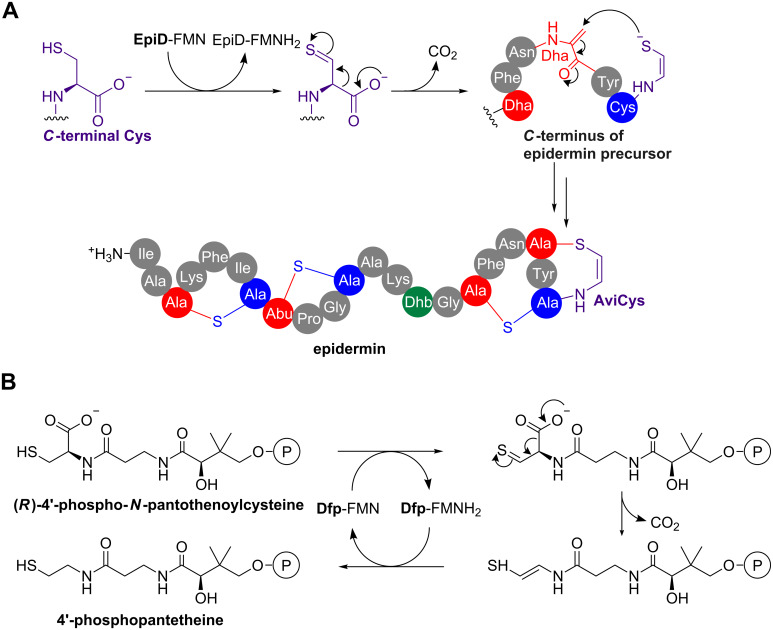
*S*-[(*Z*)-2-Aminovinyl]-D-cysteine (AviCys) formation in the epidermin pathway. A) Mechanisms for decarboxylation and 1,4-addition. B) Mechanism for the *E. coli* Dfp-catalysed conversion of (*R*)-4'-phospho-*N*-pantothenoylcysteine into 4'-phosphopantetheine during coenzyme A biosynthesis. The function of Dfp was discovered following the mechanistic characterisation of EpiD.

A structural variation on the lanthionine linkage is the C-terminal aminovinylcysteine [62] (AviCys, Figure 5A). This is found in a variety of RiPPs that also feature conventional lanthionine rings, such as epidermin [63] (Figure 5A), mersacidin [64] and cypemycin [65]. In epidermin, a *S*-[(*Z*)-2-aminovinyl]-D-cysteine (AviCys) residue is formed by the 1,4-nucleophilic addition of an oxidatively decarboxylated cysteine residue onto a Dha residue derived from serine (Figure 5A). Extensive in vitro experiments indicate that decarboxylation of cysteine precedes 1,4-addition and is catalysed by a flavoprotein (EpiD) in epidermin biosynthesis [66–67], which uses flavin mononucleotide (FMN) to oxidise the cysteine. A mechanistic proposal based on structural data involves the oxidation of the thiol to a thioaldehyde, which then functions as an electron sink to facilitate decarboxylation to generate the double bond between C_α_ and C_β_ [66] (Figure 5A). The functional characterisation of EpiD led to the identification of homologous bacterial flavoproteins (Dfp) that catalyse the decarboxylation of 4’-phospho-*N*-pantothenoylcysteine to 4’-phosphopantetheine, which is essential for coenzyme A biosynthesis [68] (Figure 5B). This demonstrates how the mechanistic analysis of secondary metabolism can inform the characterisation of primary metabolism. Surprisingly, the gene cluster for the AviCys-containing RiPP cypemycin indicates that this pathway features an alternative way to produce dehydrated amino acids [65]. Firstly, the cluster does not encode any Lan-like dehydratases, and secondly, the Dha residue required for AviCys formation derives from cysteine rather than serine.

### Pyridine and piperidine formation in thiopeptides

**Figure 6:**
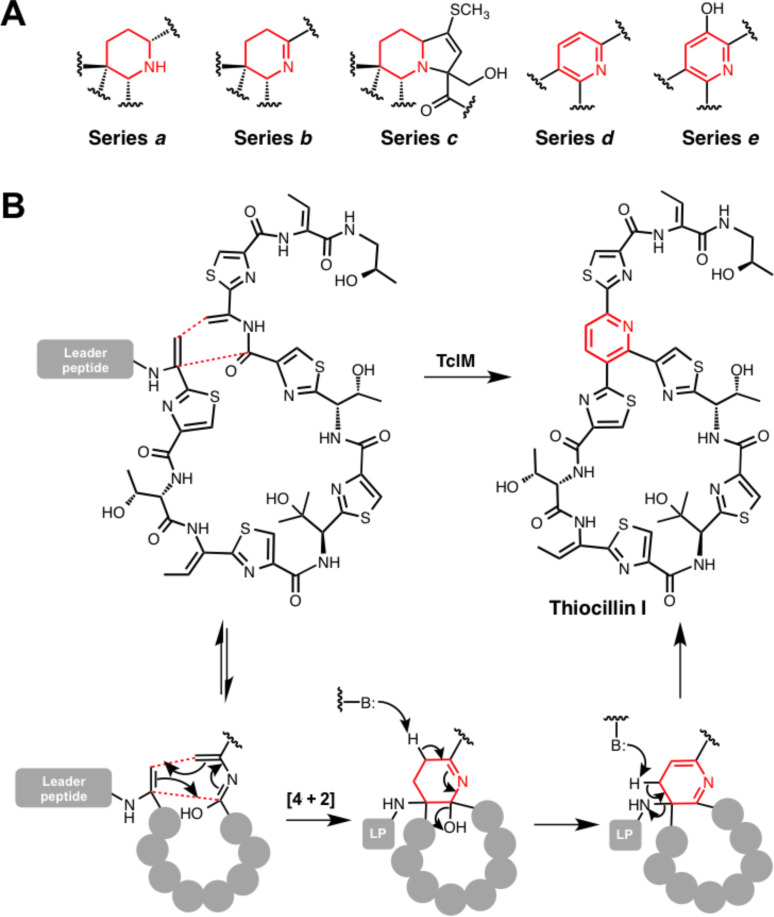
Cyclisation in the biosynthesis of thiopeptides. A) Mechanism of TclM-catalysed heterocyclisation in the biosynthesis of thiocillin I. B) An overview of the various 6-membered nitrogen-containing heterocycles that are found in thiopeptides.

Thiopeptides are a widespread bacterial RiPP family that are characterised by multiple thiazoles, dehydrated residues and a central substituted pyridine, dehydropiperidine or piperidine ring [69] (Figure 6A). Micrococcin was the first member to be identified [70], while the most well-studied member of the class is thiostrepton [71], whose gene cluster was the first of this class to be reported [72–73], along with the thiocillin and siomycin A gene clusters [73–74]. Thiopeptides are antibacterial towards Gram-positive species by inhibiting protein biosynthesis [75], but some members also exhibit biological activity towards a number of eukaryotic targets, which makes them promising anticancer [76–77] and antimalarial [78] compounds. Intriguingly, a recent study identified actively transcribed thiopeptide gene clusters in human microbiota from every body site assessed [6].

Thiazoles in thiopeptides are introduced by a BCD-protein system described previously, while threonine and serine residues are dehydrated by lantibiotic-like dehydratases. The formation of core pyridine, dehydropiperidine or piperidine is consistent with a [4 + 2] cycloaddition across two dehydrated serine residues [79–80]. Genetic disruption of *tclM* from the thiocillin pathway showed that TclM was responsible for this transformation [81], although the precise cyclisation mechanism (concerted or stepwise) could not be distinguished. Therefore, a synthetic peptide substrate was tested with recombinant TclM [82]. This showed that standalone TclM does function as a “hetero-Diels–Alderase” and a potential concerted mechanism has been proposed that involves the imidic acid tautomer of one amino acid residue (Figure 6B). The enzyme is also capable of catalysing aromatisation by elimination of water and the leader peptide. Aromatisation via leader peptide elimination does not happen in the biosynthesis of various thiopeptides, including thiostrepton, which indicates that TclM could have an active role in this elimination step.

### Macrolactam and macrolactone formation

A diverse array of macrolactams are found in RiPPs from bacteria [31], plants [83] and mammals [84]. These can arise from a variety of routes: (i) head-to-tail cyclisation by attack of the N-terminal amine of the core peptide onto the C-terminus [85]; (ii) attack of a side-chain amine onto a carbonyl [86]; (iii) condensation between the N-terminal amine of the core peptide onto a side-chain carboxylate [87]. Biochemically, these macrolactams are formed via two distinct routes: (a) ATP-dependent activation of carboxylates [88], and (b) peptidase-like cyclisation onto internal amides [85].

#### (a) ATP-dependent macrolactam and macrolactone formation

**Figure 7:**
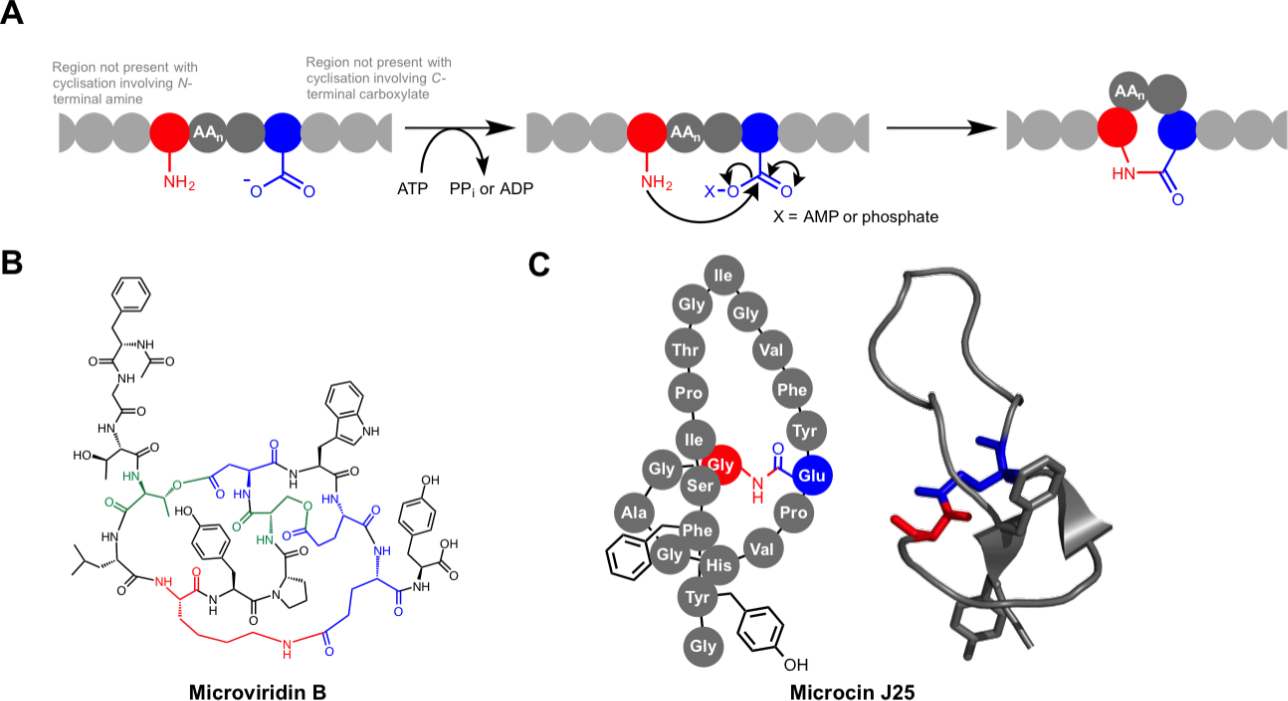
ATP-dependent macrocyclisation. A) General mechanism for ATP-dependent macrolactonisation or macrolactamisation in RiPPs. B) Structure of microviridin B, where the nucleophilic residues involved in the formation of cyclic esters are coloured green. C) Illustration of microcin J25 alongside a solution NMR structure [93] of this molecule (PDB: 1PP5). The Phe19 and Tyr20 side chains are shown in both structures to illustrate how the lasso peptide is conformationally restricted following cyclisation.

ATP-dependent macrolactam formation occurs in the biosynthesis of the lasso peptides [87] and the microviridins [86,89] (Figure 7). Lasso peptides are bacterial RiPPs that are characterised by their knotted structures, where a tail peptide is threaded through a macrolactam that is formed by the condensation of the N-terminal amino group with an asparatate or glutamate side-chain carboxylate. These are highly stable structures, and lasso peptides with a variety of biological activities have been identified [87,90]. The most well-studied member of the family is microcin J25 (Figure 7C) from *E. coli* AY25*.* Initial structural characterisation incorrectly identified microcin J25 as a conventional head-to-tail macrocyclic peptide [91], which was later revised to the lassoed structure by multiple groups [92–94]. McjC was identified as the macrolactam synthetase using both genetic inactivation in *E. coli* and in vitro analysis of purified protein [95]. McjC has homology to asparagine synthetases and the reaction they catalyse is mechanistically similar [96], although McjC lacks the N-terminal domain that catalyses the hydrolysis of glutamine to glutamic acid and ammonia [95]. The McjB peptidase first removes the leader peptide to expose an N-terminal amino group, which is usually a glycine residue, although other residues have been identified at this position [97–98]. McjC then catalyses cyclisation by activating the carboxylate of an aspartate or glutamate side chain at position 7, 8 or 9 using ATP. This generates an acyl-AMP intermediate, which is then attacked by the α-NH_2_ group of the N-terminal amino acid to form the isopeptide bond. Crucially, the precursor peptide is pre-folded so that once the lactam is formed the C-terminal tail is trapped within the macrolactam due to the position of bulky side chains on the lasso peptide tail (Phe19 and Tyr20, Figure 7C).

Microviridins constitute a much smaller family of RiPPs than lasso peptides and have only been identified from a limited number of cyanobacteria [99–103]. Members of this family can feature both macrolactams and macrolactones, and both of these are introduced by ATP-grasp ligases [88]. These macrolactams are formed by the condensation between the side chains of lysine and glutamate residues, whereas the macrolactones form from the condensation of threonine or serine side chains with aspartate or glutamate side chains. Studies on microviridin B (Figure 7B) from *Microcystis aeruginosa* NIES298 and microviridin K from *Planktothrix agardhii* CYA126/8 demonstrated that one ligase is responsible for ester formation and another catalyses amide formation [86,89]. In vitro studies on the microviridin K pathway showed that one ATP-grasp ligase catalyses the formation of two macrolactone rings, which precedes macrolactam formation [88]. The stoichiometric generation of phosphate during lactonisation indicates that the acid side chains are activated as carboxylate-phosphate mixed anhydrides, which are then attacked by serine or threonine to release phosphate. The sequence similarity between the ligases in the microviridin pathway points towards an equivalent mechanism for lactam formation, although this has not yet been demonstrated experimentally.

#### (b) Peptidase-like macrolactam formation

**Figure 8:**
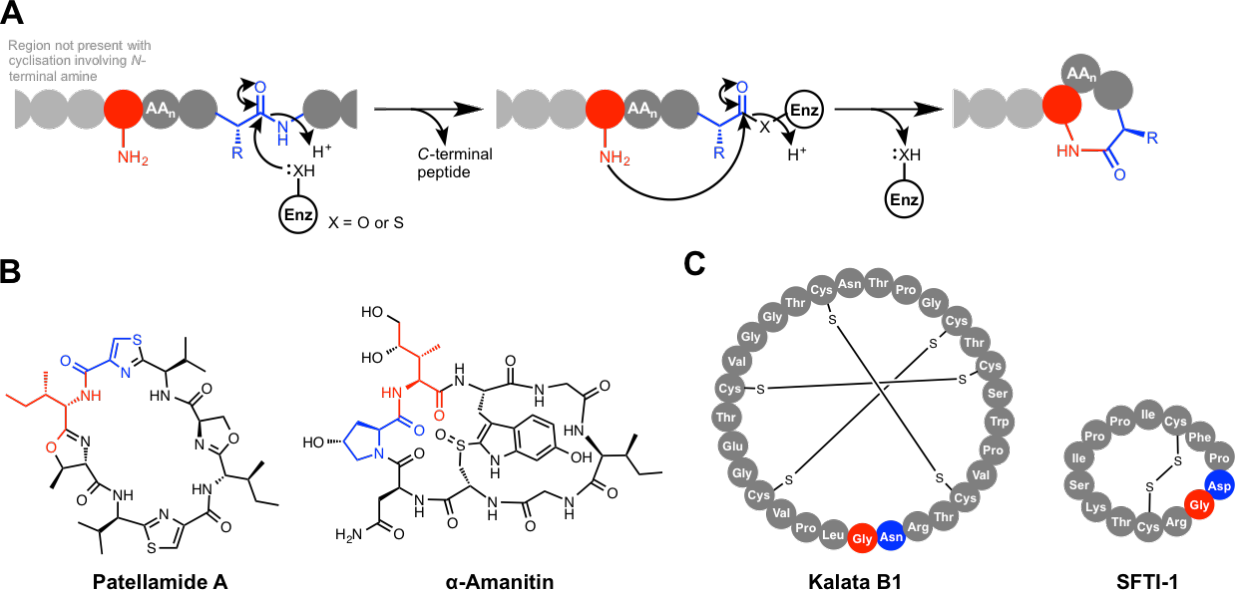
Peptidase-like macrolactam formation. A) General mechanism. B) Examples of RiPPs cyclised by serine protease-like enzymes. C) Examples of RiPPs cyclised by cysteine protease-like enzymes.

An alternative route to macrolactams involves the use of protease-like proteins that catalyse cyclisation via a ping-pong mechanism [85,104–105] (Figure 8A). In fact, protease-mediated ligation is a well-established concept and early studies showed that peptide bond formation could be achieved by modulating protease reaction conditions accordingly [106]. This has since been found to happen in the biosynthesis of cyclic RiPPs from a wide range of hosts, including cyclic peptides from both plants [105] and bacteria [104] (Figure 8B and C). Mechanistically, these cyclases function in an analogous way to either cysteine proteases or serine proteases, and these RiPP cyclases often belong to these peptidase superfamilies. The PatG cyclase from the patellamide cyanobactin pathway [31] has been very well characterised to show that one of its domains (PatGmac) possesses similarity to subtilisin-like peptidases [104,107]; accordingly, this catalyses macrocyclisation via a serine protease-like mechanism. PatG features a canonical serine protease-like catalytic triad (Asp548, His618 and Ser783), which cuts before an AYDG motif on the precursor peptide. This generates an acyl–enzyme intermediate, where the C-terminus of the peptide is bound to Ser783 as an ester. The N-terminal amino group then attacks this intermediate to generate a cyclic octapeptide. This is mechanistically similar to thioesterase-catalysed macrocyclisation found in NRP biosynthesis, although the energetic demands of breaking an amide bond versus a thioester bond are notably different. PatG may have synthetic utility, as studies with unnatural substrates have shown that macrocycles of between 5–22 residues can be produced [108], despite it naturally producing a cyclic octapeptide.

A serine protease-like cyclase (PCY1) is also found in the biosynthesis of Caryophyllaceae-type cyclic peptides in *Saponaria vaccaria* [109]*.* This cyclase functions in an analogous way to PatG, although PCY1 has structural similarity to S9a family serine peptidases, whereas PatG belongs to the S8 family. Another S9a family serine protease-like cyclase features in the biosynthesis of α-amanitin (Figure 8B), an amatoxin produced by the fungus *Amanita phalloides* and related fungi [110]. Amatoxins are responsible for many of the fatalities caused by mushroom poisoning of humans, where they function by inhibiting RNA polymerase II [111]. In the α-amanitin pathway [112], a prolyl oligopeptidase-like enzyme catalyses both hydrolysis of the leader peptide and transpeptidation to yield a backbone macrolactam [113]. No distinguishing features have been identified to indicate how it preferentially catalyses cyclisation over hydrolysis.

Given the discovery of serine protease-like cyclisation in RiPP biosynthesis, it is not surprising that cysteine protease-like enzymes have also evolved the ability to cyclise ribosomal peptides. A well-characterised cysteine protease-like macrocyclase is found in the biosynthesis of the 14-residue sunflower trypsin inhibitor 1 (SFTI-1, Figure 8C), where asparaginyl endopeptidase (AEP) employs a catalytic triad of Asn, His and Cys to catalyse both proteolysis and cyclisation [105,114–115]. SFTI-1 is found in sunflower seeds and its precursor peptide, prealbumin, is processed into both SFTI-1 and albumin [115]. Evidence towards the mechanism of AEP-catalysed cyclisation was provided by an in situ assay that used the enzyme isolated from sunflower seeds [115]. This showed that the enzyme is directly responsible for cyclisation and that the reaction does not involve full hydrolysis of the precursor peptide; this indicates that it catalyses cyclisation by amine attack onto an acyl–enzyme intermediate. Furthermore, AEP is a broad specificity peptidase that can also catalyse regular peptide hydrolysis, including excision of the SFTI-1 core peptide from prealbumin. This means that macrolactam formation is somewhat inefficient and a significant amount of acyclic SFTI-1 is also produced, but this is masked by the rapid in vivo degradation of this unwanted side-product [105].

Gene silencing experiments have linked AEP-like proteins to the macrocylisation of other cyclic plant RiPPs, including kalata-type cyclotides [85] (Figure 8C) and cyclic knottins [116], especially because the ligation site almost always features an Asx residue. *Clitoria ternatea* is a tropical plant that produces cylotides, and a remarkably efficient peptide ligase, butelase 1, was identified from this plant that is capable of cyclising a range of native and non-native peptides of between 14 to 58 residues [117]. This enzyme belongs to the AEP family, but in contrast to sunflower seed AEP, it preferentially catalyses cyclisation over hydrolysis and is actually the fastest known peptide ligase or cyclase. The variety of unrelated cyclic peptides from phylogenetically distant plant families that are processed by AEP family proteins has led to the theory that this reflects evolutionary parallelism, where AEP functions as a constraining evolutionary channel due to its capacity to catalyse cyclisation [116]. Butelase 1 can also catalyse peptide ligation when a short C-terminal sequence motif of NHV is used as the acceptor, where N is the site of ligation. Conversely, the well-characterised peptide ligase sortase A (SrtA) has been employed to catalyse cyclisation using a cysteine protease-like mechanism [118]. In vivo, this staphylococcal protein ligates proteins with a C-terminal LPXTG motif to the peptidoglycan, via the formation of an enzyme bound thioester on the threonine residue, and has been used widely as an enzymatic tool for ligation to proteins with an LPXTG tag. Cyclisation can be achieved using SrtA by the same principle, although this does require oligo-Gs at the N-terminus for efficient cyclisation [119].

**Figure 9:**
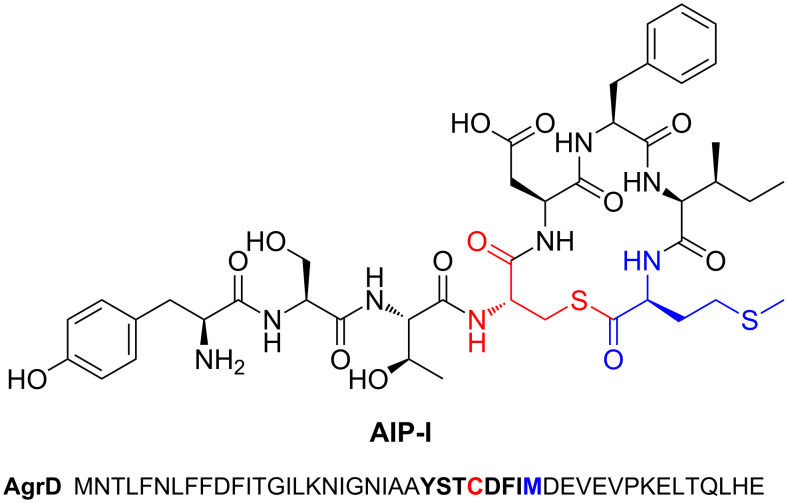
Structure of autoinducing peptide AIP-I from *Staphylococcus aureus* and the sequence of the corresponding precursor peptide AgrD.

A cysteine protease-like cyclase is proposed in the biosynthesis of autoinducing peptide [120] (AIP). However, its function differs from the above pathways as a thiolactone is generated in AIP biosynthesis (Figure 9). Autoinducing peptides are secreted molecules that form part of a quorum-sensing system in *Staphylococcus* [121]. Heterologous expression in *E. coli* showed that only AgrD (precursor peptide) and AgrB (peptidase) are required for AIP biosynthesis, although AgrD contains an N-terminal signal peptide that is cleaved by an endogenous peptidase [120]. Unlike other macrocyclisation peptidases, AgrB does not belong to a well-characterised peptidase family, but mutagenesis experiments on Cys86 infer that a cysteine protease-like mechanism acts to generate a thioester acyl–enzyme intermediate that is then attacked by Cys28 of AgrD to generate a 16-membered thiolactone [120] (Figure 9).

### Radical SAM-catalysed oxidative cross-linking

**Figure 10:**
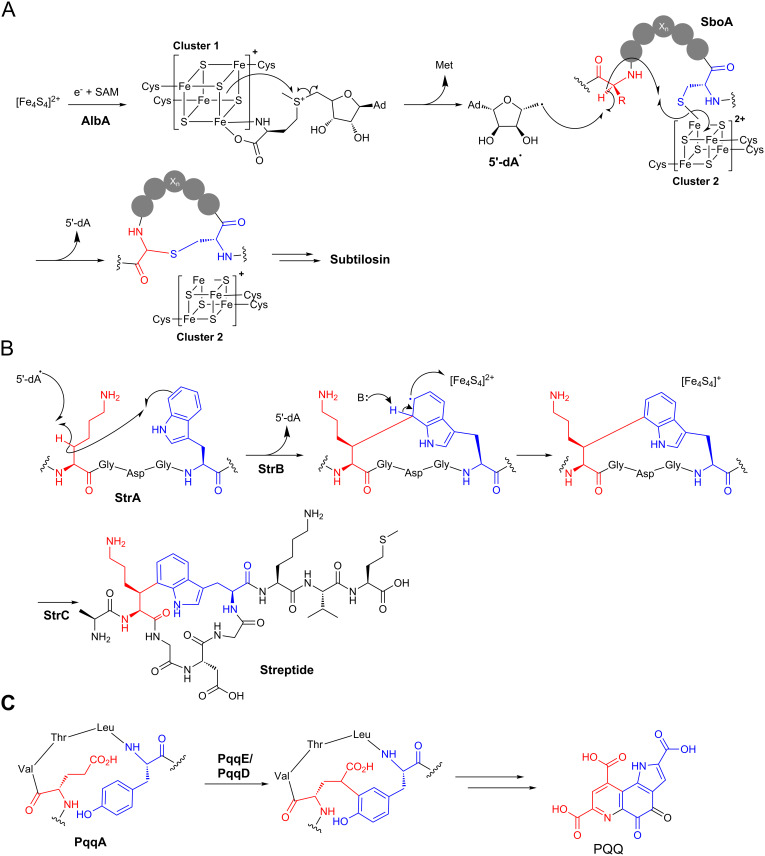
Radical cyclisation in RiPP biosynthesis. A) AlbA-catalysed formation of thioethers in the biosynthesis of subtilosin. The mechanism for deoxyadenosine radical formation is consistent throughout most radical SAM enzymes. B) Mechanism of carbon–carbon cross-linking in streptide biosynthesis. C) Proposed carbon–carbon bond formation by SPASM protein PqqE in the biosynthesis of pyrroloquinoline quinone (PQQ).

The majority of characterised cyclic RiPPs are generated by standard ionic reactions. In contrast, radical mechanisms permit reactions between unactivated atoms [122], and this exotic chemistry is employed in a number of RiPP cyclisations. In each case, cyclisation is catalysed by members of the SPASM protein family [123–124]. These are radical SAM (*S*-adenosylmethionine) proteins that contain two [4Fe–4S] binding domains, and the highly reactive iron–sulphur clusters in these proteins make them capable of carrying out complex oxidative chemistry. This protein family has been named after currently characterised pathways (SPASM = subtilosin, PQQ, anaerobic sulfatase, and mycofactocin), although the mycofactocin pathway has only been described bioinformatically [123]. Subtilosin is a *Bacillus* RiPP antibiotic that belongs to the sactipeptide family of natural products that are defined by the presence of one or more sulphur to α-carbon bonds [125]. Three thioethers in subtilosin are formed by a single SPASM protein, AlbA, and a mechanism was proposed by the Marahiel group based following detailed in vitro studies [26] (Figure 10A). The first [4Fe–4S] cluster accepts an electron from an external source to generate an active reduced form. This electron is transferred to a coordinated SAM, which is reductively cleaved to generate a 5’-deoxyadenosyl radical (5’-dA^•^). The formation of 5’-dA^•^ is common to all radical SAM proteins. The second [4Fe-4S] cluster coordinates the peptide substrate via a deprotonated thiol group of a cysteine. The 5’-dA^•^ abstracts a hydrogen from a specific α-carbon, which then attacks the thiol bound to the second [4Fe–4S] cluster. To facilitate sulphur to α-carbon bond formation, the second cluster accepts an electron. It is possible that the electron accepted by the second [4Fe–4S] cluster can be transferred to the first cluster by intramolecular electron channeling to convert both clusters into their active forms. A study on thioether bond formation during the biosynthesis of sporulation killing factor, another *Bacillus* sactipeptide, was in agreement with this mechanistic model [126].

Another SPASM protein involved in RiPP cyclisation is found in the biosynthesis of streptide, a streptococcal RiPP that is involved in bacterial communication [127]. Here, StrB catalyses the formation of a carbon–carbon bond between lysine and tryptophan side chains [25]. This is proposed to be mechanistically similar to thioether bond formation, although the role of the second [4Fe–4S] cluster is likely to differ slightly as it is unlikely that either carbon initially bonds to this cluster (Figure 10B). Instead, a radical on the lysine β-carbon (generated by 5’-dA^•^ hydrogen abstraction) attacks C-7 on the tryptophan ring. This generates an indolyl radical that can lose an electron to the second [4Fe–4S] cluster along with simultaneous loss of a proton to rearomatise. An analogous reaction takes place in the biosynthesis of the bacterial cofactor pyrroloquinoline quinone (PQQ), where the SPASM protein PqqE was proposed to catalyse the oxidative cross-linking of carbon bonds on glutamate and tyrosine side chains [17] (Figure 10C). This proposal was confirmed by in vitro reconstitution of PqqE activity with PqqA [128]. Interestingly, PqqE activity is dependent on PqqD, a 10 kDa protein that functions as a chaperone that tightly binds PqqA [129]. This key interaction promotes an association with PqqE, which then catalyses cross-linking. A number of SPASM proteins actually have a PqqD-like domain at their N-terminus, including AlbA and ThnB [130]. ThnB catalyses thioether bridge formation in thurincin H biosynthesis, and in vitro analysis demonstrated that its PqqD-like domain is essential for catalysing thioether formation, but not for SAM cleavage activity [130].

### Notable uncharacterised RiPP cyclisations

**Figure 11:**
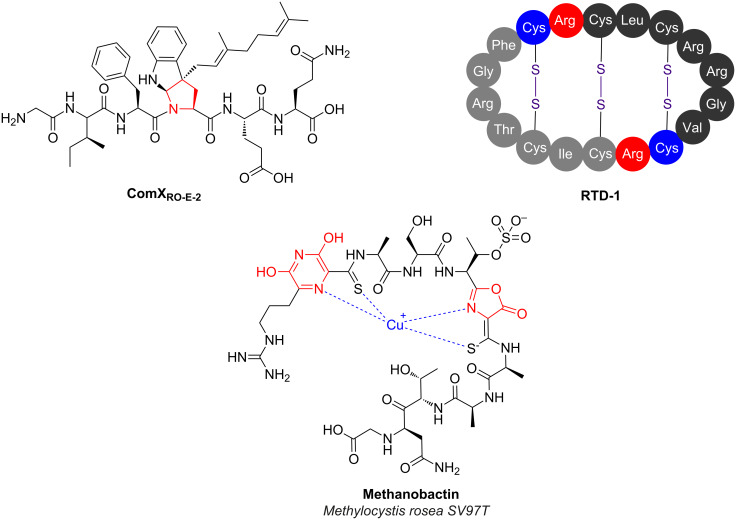
RiPPs with uncharacterised mechanisms of cyclisation. Unusual heterocycles in ComX and methanobactin are indicated in red. RTD-1 is formed by the head-to-tail dimerisation of precursor peptides encoded on two separate genes.

Despite the huge progress that has been made over the past couple of decades on RiPP cyclisation, there still exist a number of notable pathways where key cyclisation mechanisms have not yet been determined. This is often due to the lack of suitable candidate enzymes, especially in eukaryotic pathways where gene clustering is less common. Otherwise, it could reflect the challenges associated with expression of functional soluble protein or the generation of a suitable substrate for candidate enzymes. A number of these cyclisations are found in partially characterised pathways, such as the S–C cross-link in α-amanitin (Figure 8B) that is formed between cysteine and tryptophan residues (the tryptathionine linkage [131]). The ComQXPA quorum sensing (QS) system [132] found in *Bacillus* species represents another partially characterised pathway that features an unusual cyclised RiPP [133]. Mature ComX is a secreted RiPP that functions as a signal in this QS system, and the cyclised residue is crucial for its bioactivity [134]. The precursor peptide ComX is modified by a isoprenyl transferase (ComQ), which transfers an isoprenyl group to position 3 of the indole side chain of a conserved tryptophan residue [135]. This directly generates a tricyclic structure, presumably via attack of the main chain amide nitrogen onto the iminium intermediate that is generated following prenylation (Figure 11).

#### Class IV bacteriocins

Class IV bacteriocins are a broad class of cyclic bacterial RiPP where their N- and C-termini are linked by a peptide bond [136]. This broad class consists of globular, α-helical and thermostable cyclic peptides, and includes molecules whose pathways are poorly understood, such as enterocin AS-48 [137–138] (also known as bacteriocin 21 [139]). AS-48 is a 70-residue cyclic antibiotic produced by *Enterococcus faecalis* and was recently shown to enhance the ability of the strain to colonise the mammalian gastrointestinal tract by outcompeting bacteria that are sensitive to AS-48 [140]. A gene cluster has been identified [141], and site-directed mutagenesis has been used to identify key residues in the precursor peptide that are critical for cyclisation [142], but the actual cyclase has not been characterised. One explanation for the limited understanding of this pathway is that the leader peptide removal and cyclisation could be catalysed by membrane associated proteins (perhaps as a complex), which hinders biochemical characterisation. Alternatively, these biosynthetic proteins may exist elsewhere in the *Enterococcus* genome.

#### Defensins

Mammals produce various antimicrobial peptides (AMPs) that have important roles in the mammalian immune system [143], including in humans [144], and these AMPs often exist as a cocktail of compounds. Many of these are unmodified linear peptides, such as the human peptide cathelicidin LL-37 [145], or are cyclised by disulphide bonds, such as human β-defensin hBD-2 [146]. However, there is one class of backbone-cyclised AMP in mammals, the θ-defensins [84]. These are found in Old World monkeys and orangutans, but are not made by New World monkeys or humans. θ-Defensins, such as RTD-1 (Figure 11), are 18-residue peptides that are formed by the head-to-tail cyclisation of two nonapeptides that are themselves derived from the C-terminal region of precursor peptides, and both heterodimers or homodimers can be formed in this process [84,144]. Along with their antimicrobial activity, these peptides can inhibit fusion of HIV-1 to host cells [147]. Surprisingly, the human genome contains six θ-defensin pseudogenes that are actually expressed [148]. However, these contain premature stop codons that prevent the proper expression of these precursor peptides. Remarkably, aminoglycoside-induced stop codon readthrough of these genes in human-tissue cultures leads to the production of properly cyclised θ-defensins that possess antimicrobial activity [148], indicating that humans have retained the proteins required for processing and cyclisation. The identity of these genes in either humans or monkeys has not been found, although a peptidase-like mechanism can be speculated.

#### Methanobactins

Methanobactins are copper-binding RiPPs produced by methanotrophic bacteria [149–150]. A methane monooxygenase (MMO) used by these bacteria requires copper as a cofactor, so the requirement for copper with these methanotrophs is much higher than in other bacteria [151]. Therefore, methanobactins assist with copper uptake for these bacteria and have been shown to participate in the control of the “copper-switch” that regulates whether copper-containing or copper-free MMO is expressed [152]. Thus far, methanobactins have been identified that contain oxazolones and pyrazinediones [150,153–154], which are found alongside thioamides in these molecules (Figure 11). These post-translational modifications are critical for copper binding but the mechanisms of these heterocyclisation steps have not yet been determined for any pathway, despite the identification of various gene clusters [150,154]. A bioinformatic analysis showed that methanobactin-like pathways are found in non-methanotrophic bacteria [154], although the products and roles of these gene clusters are currently unknown.

### Future challenges

There have been stunning advances in the discovery and characterisation of RiPP post-translational modifications in recent years [5,14]. Much of this has been led by genomics, which has informed both the study of established molecules whose biosynthetic origins were previously unknown (e.g., thiostrepton [72]) and the discovery of new pathways via genome mining [155–157]. However, gene cluster identification does not provide detailed mechanistic information about post-translational modifications and there are numerous examples where key steps in pathways with sequenced gene clusters have not been characterised (see examples above). More widely, it is clear that there are a vast number of uncharacterised pathways encoded in sequenced genomes [8,158]. Many of these are homologous to known RiPP classes, such as uncharacterised lasso peptide and lanthipeptide pathways that are highly prevalent in many bacterial genomes [49,87,159], although it is evident that many novel classes of RiPP await characterisation [7].

Despite the successes reported above, genome mining for novel RiPP clusters is hindered by a number of factors. Firstly, RiPP “gene clusters” can be as small as two genes: a precursor peptide and a tailoring protein, especially when further hydrolytic processing can be carried out by endogenous peptidases [120]. The prevalence of putative small peptides encoded throughout genomes [160] make it difficult to predict which of these are post-translationally processed, and some small genes are overlooked by automated gene annotation software, which means that some putative RiPP precursors are not even listed in databases. Furthermore, novel classes are difficult to identify precisely due to their novelty compared to known pathways. This is in contrast to terpenes, polyketide synthases or NRPSs, whose pathways are all clearly identified by signature protein domains. Finally, many RiPPs do not possess antimicrobial or cytotoxic activity, so are not identified by classical activity-based screens.

Mass spectrometry (MS) represents a relatively unbiased approach to screening for the production of novel RiPPs, although this is non-trivial due to the variety of unusual post-translational modifications that could take place. This means that product masses and fragmentation patterns are very difficult to predict, especially when peptides are cyclised [161]. Despite these issues, significant progress has been made to develop methods to correlate MS data with RiPP genomic data [162], although these methods still have focused on known RiPP classes with relatively predictable modifications [155–157,161]. The use of ultra-tolerant search terms does allow for the identification of peptides with unexpected post-translational modifications [163], although this method has not been applied to bacterial RiPPs.

To overcome these barriers to discovery, various search algorithms have been developed or adopted to identify truly novel RiPP gene clusters. For example partial phylogenetic profiling was used to propose the currently uncharacterised “mycofactocin” family of gene clusters [123]. A similar approach was also used to propose a family of selenocysteine-containing RiPPs [18]. An alternative approach is to screen for homology to tailoring proteins from known pathways, which can be particularly effective when RiPP-specific protein classes are assessed. For example, thousands of gene clusters with limited homology to TOMMs were identified by searching for clusters associated with YcaO domain proteins [43,164], which are essential for heterocyclisation. These pathways may have some mechanistic similarities with known TOMM pathways, but the diversity of precursor peptide sequences identified, along with novel combinations of predicted tailoring enzymes, indicates that the products of these pathways will be significantly different to known RiPPs. Similar results were obtained when mining for lanthipeptide-like gene clusters [7,53], and widespread searches for pathways with RiPP-like tailoring enzymes can be carried out using BAGEL3 [9]. More generally, a hidden Markov model-based probabilistic algorithm, ClusterFinder, identified hundreds of putative new classes of RiPP alongside novel clusters for the biosynthesis of other natural product classes [8,158]. These bioinformatic analyses all indicate that a vast amount of the RiPP landscape remains unexplored, and a major future challenge will be to determine the both identity and the biological function of these putative metabolites.

## Conclusion

A remarkable array of RiPP cyclisation steps have been identified and subsequently mechanistically characterised. These biosynthetic steps enable producing organisms to convert simple ribosomal precursor peptides into complex molecules with exquisite biological activities. There is a degree of commonality between the modification steps that have been characterised for both RiPPs and for other secondary metabolite pathways, but it is interesting to note that there are a significant number of biochemical modifications that, thus far, appear to be unique to RiPP biosynthesis. For example, lanthionine formation, YcaO protein-catalysed heterocyclisation and radical SAM-catalysed thioether cross-links are only found in RiPP biosynthetic pathways. Much recent work on RiPP biosynthesis has been assisted by the rapid identification of gene clusters by next generation sequencing technologies, and this widespread genome sequencing also indicates that there remains a wealth of unexplored pathways to discover and characterise.
